# Supplementary material for: Designing of immunodiagnostic assay using polyclonal antibodies for detection of Enteropathogenic Escherichia coli strains
Source: PLoS One. 2024 Dec 30;19(12):e0315848. doi: 10.1371/journal.pone.0315848 (PMC11684673; doi:10.1371/journal.pone.0315848)
Supplement: Attachment — Submitted filename: Comment to authors.docx [file pone.0315848.s001.docx]

Comment to authors

I am pleased to have had the opportunity to review this manuscript due to the importance of the study, which aims to develop a diagnostic technique for E. coli, a neglected but significant bacterial pathogen. This technique could greatly improve the speed of diagnosis and treatment of E. coli infections, making it a valuable contribution to patient care.

**General comments**

#1. Given the availability of multiple advanced options in today’s world, including molecular techniques, it would be valuable for the authors to discuss the potential gaps in their study and how it compares to these more advanced diagnostic methods. A discussion of this could strengthen the manuscript by providing a clearer understanding of the study’s relevance and limitations in the context of current diagnostic advancements.

#2. The manuscript contains several typographical and formatting errors that affect its readability. I recommend addressing the spacing issues, numerical format corrections, and inconsistencies in citation and scientific name formatting, as well as correcting other similar typographical errors.

#2.1. Specific comments;

Lines 23, 36, 64, 78, 218, 430, 499, and 507: E. coli should be italics “*E. coli*”

Lines 32; 36, 233, 317, 379, and 380: Escherichia coli should be italics to “*Escherichia coli*”

Line 37: infants (Kaur and Dudeja 2023) needs spacing “infants (Kaur and Dudeja 2023)”

Line 41: centers (Dupont et al. 2016; Gismero-Ordoñez et al. 2002) needs spacing “centers (Dupont et al. 2016; Gismero-Ordoñez et al. 2002)”

Line 77: “((Gomes et al. 2016)” delete one of the brackets “(Gomes et al. 2016)”

Lines 107, 126, and 163: -20°C spacing “- 20 °C”

Line 107: laboratory(Zenebe et al. 2024 ; Wolde et al. 2022) spacing “laboratory (Zenebe et al. 2024 ; Wolde et al. 2022)”.

Line 109: Spacing needed “37 °C”

Line 109: 300ml spacing required “300 ml”

Lines 116 and 162: 4°C spacing “4 °C”

Lines 117 and118: 2ml spacing “2 ml”

Line 121: 333µl:666µl spacing “333 µl : 666 µl”

Line 124: 50ml needs spacing “50 ml”

Line 131: 5µl needs spacing “5 µl”

Line 133: 100°C needs spacing “100 °C”

Line 138: 80rpm needs spacing “80 rpm”

Line 142: 25-30gm needs spacing “25-30 gm”

Line 151: 50μl needs spacing “50 μl”

Line 155: 3ml needs spacing “3 ml”

Line 157: 100μl needs spacing “100 μl”

Line 160: to(Joyce et al. 2014) needs spacing “to (Joyce et al. 2014)”

Line 162: 4oc needs correction “4 °C”

Line 165, 201, and 208: 10^0^, 10^-1^, 10^-2^,10^-3^ 10^-4^,10^-5^ ,10^-6^, 10^-7^ , 10^-8^,10^-9^,10^-10^  should be corrected as “10^0^, 10^-1^, 10^-2^, 10^-3^, 10^-4^, 10^-5^, 10^-6^, 10^-7^, 10^-8^, 10^-9^, and 10^-10^”

Line 173: occurs(Moges et al. 2022) needs spacing “occurs (Moges et al. 174 2022)”

Line 180: 1-12min needs spacing “1-12 min”

Line 182: persisted(Moges et al. 2022) needs spacing “persisted (Moges et al. 2022)”

Line 197: Then,3 colononies needs spacing “Then, 3 colonies”

Line 195: 40µl need spacing “40 µl”

Line 197: agglutination(Danielsson and Kronvall 1974) needs spacing “agglutination (Danielsson and Kronvall 1974)”

Line 210: at37 ºC needs spacing “at 37 °C”

Line 229: 95%confidence needs spacing “95% confidence”

Line 260: 500µl needs spacing “500 µl”

Line 260: 23 mice,300 µl needs spacing “23 mice, 300 µl”

Line 261: 3ml needs spacing “3 ml”

Line 261: (15 mice) delete brackets “15 mice”

Line 262: (fig) which figure? 1, 2, or 3? I think the authors are saying figure 3.

Line 306: 10^0^ ,10^-1^ ,10^-2^ ,10^-3^ ,10^-4^ ,and 10^-5^ should be corrected as “10^0^ , 10^-1^ , 10^-2^, 10^-3^, 10^-4^, and 10^-5^”

Line 320: immunodiagnostic assay should be bold “**immunodiagnostic assay**”

Line 413: EPEC(as a minimal CFU/ml ) needs spacing “EPEC (as a minimal CFU/ml )”

Line 424: colonies/5 μL needs spacing “colonies / 5 μL”

Line 434: biomarker(Mare et al. 2021a) needs spacing “biomarker (Mare et al. 2021a)”

Line 444: antigen(overnight culture colony) needs spacing “antigen (overnight culture colony)”

Line 467: strains(Hahm and Bhunia 2006 ; Rocha et al. 2014).. needs spacing and delete one the full stops “strains (Hahm and Bhunia 2006 ; Rocha et al. 2014).”

Line 476: finding(Medina et al. 2012) needs spacing “finding (Medina et al. 2012)”

Line 489: antibody(Hampson 1991) needs spacing “antibody (Hampson 1991)”

Line 502: method( Rocha et al. 2014b) needs spacing “method ( Rocha et al. 2014b)”

Line 504: protein(Arefin et al. 2011 ; Kalambhe et al. 2017) needs spacing “protein (Arefin et al. 2011 ; Kalambhe et al. 2017)”

Line 516: 1 ⁄ 1,000,000 do you mean “10^-6^”
