# Supplementary material for: Designing of immunodiagnostic assay using polyclonal antibodies for detection of Enteropathogenic Escherichia coli strains
Source: PLoS One. 2024 Dec 30;19(12):e0315848. doi: 10.1371/journal.pone.0315848 (PMC11684673; doi:10.1371/journal.pone.0315848)
Supplement: Attachment — Submitted filename: Response to reviewers.docx [file pone.0315848.s002.docx]

**Response to reviewers’ comments**

PONE-D-24-30559

Designing of immunodiagnostic assay using polyclonal antibodies for detection of Enteropathogenic Escherichia coli strains

PLOS ONE

Dear Dr. Yinur Mengistu,

Thank you for submitting your manuscript to PLOS ONE. After careful consideration, we feel that it has merit but does not fully meet PLOS ONE’s publication criteria as it currently stands. Therefore, we invite you to submit a revised version of the manuscript that addresses the points raised during the review process.

Please submit your revised manuscript by Nov 15 2024 11:59PM. If you will need more time than this to complete your revisions, please reply to this message or contact the journal office at <a href="mailto:plosone@plos.org">plosone@plos.org. When you're ready to submit your revision, log on to https://www.editorialmanager.com/pone/ and select the 'Submissions Needing Revision' folder to locate your manuscript file.

Please include the following items when submitting your revised manuscript:

- A rebuttal letter that responds to each point raised by the academic editor and reviewer(s). You should upload this letter as a separate file labeled 'Response to Reviewers'.
- A marked-up copy of your manuscript that highlights changes made to the original version. You should upload this as a separate file labeled 'Revised Manuscript with Track Changes'.
- An unmarked version of your revised paper without tracked changes. You should upload this as a separate file labeled 'Manuscript'.

If you would like to make changes to your financial disclosure, please include your updated statement in your cover letter. Guidelines for resubmitting your figure files are available below the reviewer comments at the end of this letter.

If applicable, we recommend that you deposit your laboratory protocols in protocols.io to enhance the reproducibility of your results. Protocols.io assigns your protocol its own identifier (DOI) so that it can be cited independently in the future. For instructions see: <https://journals.plos.org/plosone/s/submission-guidelines#loc-laboratory-protocols>. Additionally, PLOS ONE offers an option for publishing peer-reviewed Lab Protocol articles, which describe protocols hosted on protocols.io. Read more information on sharing protocols at <https://plos.org/protocols?utm_medium=editorial-email&utm_source=authorletters&utm_campaign=protocols>.

We look forward to receiving your revised manuscript.

Kind regards,

Furqan Kabir
Academic Editor
PLOS ONE

**1. Response to editor's comments**

 The changes in the manuscript are tracked as like blow:

the editor’s comments are highlighted in red, while the responses to the first, second, and third reviewers’ comments are marked in yellow, grey, and green, respectively.

1. Please ensure that your manuscript meets PLOS ONE's style requirements, including those for file naming.

Author Response: Thank you for reminding us the PLOS ONE's style. We named and arranged the files according to the requirements.

2. To comply with PLOS ONE submissions requirements, in your Methods section, please provide additional information regarding the experiments involving animals and ensure you have included details on (1) methods of sacrifice, (2) methods of anesthesia and/or analgesia, and (3) efforts to alleviate suffering.

Author Response: We thank you the editor for this important request. We incorporated a brief method of sacrifice, anesthesia and effort to reduce pain as indicated in the methodology section of the manuscript (from line 157-168).

3. Please note that funding information should not appear in any section or other areas of your manuscript. We will only publish funding information present in the Funding Statement section of the online submission form. Please remove any funding-related text from the manuscript.

Author Response: Thank you for this important information. We removed all funding-related text from the manuscript, per your request.

4. Thank you for stating the following financial disclosure: “This study was supported by the Bio and Emerging Technology Institute (BETin), Prof Tesfaye Sisay Tessema won research grant.”

Please state what role the funders took in the study. If the funders had no role, please state: "The funders had no role in study design, data collection and analysis, decision to publish, or preparation of the manuscript." If this statement is not correct you must amend it as needed. Please include this amended Role of Funder statement in your cover letter; we will change the online submission form on your behalf.

Author Response: Thank you for this important information. We included the amended role of funder statement as: "The funders had no role in study design, data collection and analysis, decision to publish, or preparation of the manuscript."

5. Please provide a complete Data Availability Statement in the submission form, ensuring you include all necessary access information or a reason for why you are unable to make your data freely accessible. If your research concerns only data provided within your submission, please write "All data are in the manuscript and/or supporting information files" as your Data Availability Statement.

Author Response: Thank you for reminding us: We revised the Data Availability Statement as "All data are in the manuscript”.

6. When completing the data availability statement of the submission form, you indicated that you will make your data available on acceptance. We strongly recommend all authors decide on a data sharing plan before acceptance, as the process can be lengthy and hold up publication timelines. Please note that, though access restrictions are acceptable now, your entire data will need to be made freely accessible if your manuscript is accepted for publication. This policy applies to all data except where public deposition would breach compliance with the protocol approved by your research ethics board. If you are unable to adhere to our open data policy, please kindly revise your statement to explain your reasoning and we will seek the editor's input on an exemption. Please be assured that, once you have provided your new statement, the assessment of your exemption will not hold up the peer review process.

Author response: We appreciate the editor; we included the data availability statements as "All authors have confirmed data availability."

7. Please include a copy of Table 4 which you refer to in your text on page 16.

Author response: We apologize for the oversight. A copy of Table 4, as referred to on page 16(399), is included in the revised manuscript.

8. Please review your reference list to ensure that it is complete and correct. If you have cited papers that have been retracted, please include the rationale for doing so in the manuscript text, or remove these references and replace them with relevant current references. Any changes to the reference list should be mentioned in the rebuttal letter that accompanies your revised manuscript. If you need to cite a retracted article, indicate the article’s retracted status in the References list and also include a citation and full reference for the retraction notice.

Author Response: We acknowledge the editor for emphasizing on the accuracy of references. We carefully reviewed and updated our reference list, correcting typographical errors and ensuring formatting consistency. We added four new references (Okeke, I. N., 2009; Belete et al., 2022; Yinur et al 2023; Govindarajan et al., 2024), replaced older references (Hampson, 1991, with Farhan and Khan, 2023; Danielson and Kronvall, 1974, with Rastawicki, 2018), and removed one outdated reference (Leenars).

Reviewers' comments:

Reviewer's Responses to Questions

**Comments to the Author**

1. Is the manuscript technically sound, and do the data support the conclusions?

The manuscript must describe a technically sound piece of scientific research with data that supports the conclusions. Experiments must have been conducted rigorously, with appropriate controls, replication, and sample sizes. The conclusions must be drawn appropriately based on the data presented.

Reviewer #1: Partly

Reviewer #2: Yes

Reviewer #3: Yes

2. Has the statistical analysis been performed appropriately and rigorously?

Reviewer #1: I Don't Know

Reviewer #2: I Don't Know

Reviewer #3: Yes

3. Have the authors made all data underlying the findings in their manuscript fully available?

The [PLOS Data policy](http://www.plosone.org/static/policies.action#sharing) requires authors to make all data underlying the findings described in their manuscript fully available without restriction, with rare exception (please refer to the Data Availability Statement in the manuscript PDF file). The data should be provided as part of the manuscript or its supporting information, or deposited to a public repository. For example, in addition to summary statistics, the data points behind means, medians and variance measures should be available. If there are restrictions on publicly sharing data—e.g. participant privacy or use of data from a third party—those must be specified.

Reviewer #1: No

Reviewer #2: Yes

Reviewer #3: Yes

4. Is the manuscript presented in an intelligible fashion and written in standard English?

PLOS ONE does not copyedit accepted manuscripts, so the language in submitted articles must be clear, correct, and unambiguous. Any typographical or grammatical errors should be corrected at revision, so please note any specific errors here.

Reviewer #1: No

Reviewer #2: Yes

Reviewer #3: Yes

5. Review Comments to the Author

Please use the space provided to explain your answers to the questions above. You may also include additional comments for the author, including concerns about dual publication, research ethics, or publication ethics. (Please upload your review as an attachment if it exceeds 20,000 characters)

**2. Response to reviewers' comments**

Reviewer #1: Immune diagnosis using polyclonal sera does not give any novelty in diagnosis of bacterial diseases. Authors used crude antigen for raising hyperimmune sera, which can always invite nonspecific/false diagnosis of bacterial diseases.
Suggestion
1. Better to go for recombinant protein based diagnosis

Authors response: We appreciate this critical insight. Due to budget constraints, the objective of this study was to develop an assay utilizing crude antigen. Nevertheless, recombinant protein-based assays would improve diagnostic specificity and loftier than crude antigen to increase the efficiency of the assay. In future work, we will explore recombinant antigen-based methods and monoclonal antibodies to further enhance assay precision.

2. It is necessary to validate the assay with reference methods

Authors response: We appreciate reviewer for pointing out this issue, and we apologize for any lack of clarity in our initial explanation. We would like to underscore that the bacterial isolates were already validated by PCR method before performing of the diagnostic performance assay test as mentioned in the methodology part of the manuscript (line 103-110).

3. Need to illustrate relative accuracy, specificity, sensitivity of the assay.

Author response: We thank you the reviewer. Sensitivity, specificity and relative accuracy are indicated on table 4 of manuscript, which was calculated using diagnostic performance matrix (MedCalc Statistical Software version 18.11.6).

Reviewer #2: The manuscript demonstrates a well-organized structure and a lucid presentation of the authors' research on the development of an immunodiagnostic assay for detecting EPEC strains. It provides a detailed description of the methods used and evaluates the assay's specificity and sensitivity across different bacterial strains.

• The manuscript has some areas that could be improved.
• It would be beneficial to have a more detailed discussion of the study's limitations, such as the potential drawbacks of using a polyclonal antibody and the likelihood of cross-reactivity with other bacterial strains. Additionally, a more comprehensive description of the statistical analysis used to assess the assay's specificity and sensitivity would enhance the manuscript.
• The figures and tables could also be improved for better clarity and readability. Furthermore, a more detailed discussion of the potential applications of the assay in clinical and public health settings would be valuable.

Author response: We are grateful for your positive feedback on the structure and clarity of our manuscript, as well as for your valuable suggestions for improvement. We have expanded the discussion to address limitations, including the risk of cross-reactivity when using polyclonal antibodies, and we have clarified the statistical analysis used. Additionally, we have discussed the broader potential applications of our assay in clinical and public health settings. Regarding the table and figures, we provided the improved table (see table 4) and figures that we have at this time.

line 103-110, we mentioned the validation of bacterial isolates by PCR.

line 446-454, we added the limitation of currently available EPEC diagnostic assays.

line 532-539, statistical analysis is clarified and elaborated.

line 561-565, we included the application and limitation of this assay in clinical setting.

Reviewer #3: Comment to authors
I am pleased to have had the opportunity to review this manuscript due to the importance of the study, which aims to develop a diagnostic technique for E. coli, a neglected but significant bacterial pathogen. This technique could greatly improve the speed of diagnosis and treatment of E. coli infections, making it a valuable contribution to patient care.
General comments
#1. Given the availability of multiple advanced options in today’s world, including molecular techniques, it would be valuable for the authors to discuss the potential gaps in their study and how it compares to these more advanced diagnostic methods. A discussion of this could strengthen the manuscript by providing a clearer understanding of the study’s relevance and limitations in the context of current diagnostic advancements.

Author response: We acknowledge the reviewer for this suggestion. In response your comments, we added a discussion comparing our immunodiagnostic approach to molecular techniques (line 446-454), acknowledging the limitations (line 561-565) and relevance of our study in light of advanced diagnostic options.

#2. The manuscript contains several typographical and formatting errors that affect its readability. I recommend addressing the spacing issues, numerical format corrections, and inconsistencies in citation and scientific name formatting, as well as correcting other similar typographical errors.

Author response: We apologize for the typographical errors present in the original manuscript. We corrected the issues you pointed out including, spacing, italicizing, and numerical formatting as citation typos throughout the manuscript.

#2.1. Specific comments;
Lines 23, 36, 64, 78, 218, 430, 499, and 507: E. coli should be italics “E. coli”
Lines 32; 36, 233, 317, 379, and 380: Escherichia coli should be italics to “Escherichia coli”
Line 37: infants (Kaur and Dudeja 2023) needs spacing “infants (Kaur and Dudeja 2023)”
Line 41: centers (Dupont et al. 2016; Gismero-Ordoñez et al. 2002) needs spacing “centers (Dupont et al. 2016; Gismero-Ordoñez et al. 2002)”
Line 77: “((Gomes et al. 2016)” delete one of the brackets “(Gomes et al. 2016)”
Lines 107, 126, and 163: -20°C spacing “- 20 °C”
Line 107: laboratory(Zenebe et al. 2024 ; Wolde et al. 2022) spacing “laboratory (Zenebe et al. 2024 ; Wolde et al. 2022)”.
Line 109: Spacing needed “37 °C”
Line 109: 300ml spacing required “300 ml”
Lines 116 and 162: 4°C spacing “4 °C”
Lines 117 and118: 2ml spacing “2 ml”
Line 121: 333µl:666µl spacing “333 µl : 666 µl”
Line 124: 50ml needs spacing “50 ml”
Line 131: 5µl needs spacing “5 µl”
Line 133: 100°C needs spacing “100 °C”
Line 138: 80rpm needs spacing “80 rpm”
Line 142: 25-30gm needs spacing “25-30 gm”
Line 151: 50μl needs spacing “50 μl”
Line 155: 3ml needs spacing “3 ml”
Line 157: 100μl needs spacing “100 μl”
Line 160: to(Joyce et al. 2014) needs spacing “to (Joyce et al. 2014)”
Line 162: 4oc needs correction “4 °C”
Line 165, 201, and 208: 100, 10-1, 10-2,10-3 10-4,10-5 ,10-6, 10-7 , 10-8,10-9,10-10 should be corrected as “100, 10-1, 10-2, 10-3, 10-4, 10-5, 10-6, 10-7, 10-8, 10-9, and 10-10”
Line 173: occurs(Moges et al. 2022) needs spacing “occurs (Moges et al. 174 2022)”
Line 180: 1-12min needs spacing “1-12 min”
Line 182: persisted(Moges et al. 2022) needs spacing “persisted (Moges et al. 2022)”
Line 197: Then,3 colononies needs spacing “Then, 3 colonies”
Line 195: 40µl need spacing “40 µl”
Line 197: agglutination(Danielsson and Kronvall 1974) needs spacing “agglutination (Danielsson and Kronvall 1974)”
Line 210: at37 ºC needs spacing “at 37 °C”
Line 229: 95%confidence needs spacing “95% confidence”
Line 260: 500µl needs spacing “500 µl”
Line 260: 23 mice,300 µl needs spacing “23 mice, 300 µl”
Line 261: 3ml needs spacing “3 ml”
Line 261: (15 mice) delete brackets “15 mice”
Line 262: (fig) which figure? 1, 2, or 3? I think the authors are saying figure 3.
Line 306: 100 ,10-1 ,10-2 ,10-3 ,10-4 ,and 10-5 should be corrected as “100 , 10-1 , 10-2, 10-3, 10-4, and 10-5”
Line 320: immunodiagnostic assay should be bold “immunodiagnostic assay”
Line 413: EPEC(as a minimal CFU/ml ) needs spacing “EPEC (as a minimal CFU/ml )”
Line 424: colonies/5 μL needs spacing “colonies / 5 μL”
Line 434: biomarker(Mare et al. 2021a) needs spacing “biomarker (Mare et al. 2021a)”
Line 444: antigen(overnight culture colony) needs spacing “antigen (overnight culture colony)”
Line 467: strains(Hahm and Bhunia 2006 ; Rocha et al. 2014).. needs spacing and delete one the full stops “strains (Hahm and Bhunia 2006 ; Rocha et al. 2014).”
Line 476: finding(Medina et al. 2012) needs spacing “finding (Medina et al. 2012)”
Line 489: antibody(Hampson 1991) needs spacing “antibody (Hampson 1991)”
Line 502: method( Rocha et al. 2014b) needs spacing “method ( Rocha et al. 2014b)”
Line 504: protein(Arefin et al. 2011 ; Kalambhe et al. 2017) needs spacing “protein (Arefin et al. 2011 ; Kalambhe et al. 2017)”
Line 516: 1 ⁄ 1,000,000 do you mean “10-6”

6. PLOS authors have the option to publish the peer review history of their article ([what does this mean?](https://journals.plos.org/plosone/s/editorial-and-peer-review-process#loc-peer-review-history)). If published, this will include your full peer review and any attached files.


If you choose “no”, your identity will remain anonymous but your review may still be made public.

Reviewer #1: No

Reviewer #2: **Yes:** Nabi Jomehzadeh

Reviewer #3: No

Thank you for your valuable feedback on our manuscript.

Regarding the notation on lines 165 and 306, we have corrected the spacing around the numeric superscripts, as recommended. However, we have retained the superscript format to clearly indicate the dilution factors, as this notation is essential for clarity in representing the dilution factors.

We hope that these revisions address the reviewers' and editor's concerns comprehensively. Thank you again for your valuable feedback and for the opportunity to improve our manuscript.
